# Mild multiple sclerosis challenges shape work experiences, affect self-concept, and are often trivialized despite disclosure

**DOI:** 10.3389/fresc.2025.1677114

**Published:** 2025-11-12

**Authors:** Maria Grytvik Hartvedt, Britt Normann, Marianne Sivertsen, Ellen Christin Arntzen

**Affiliations:** 1 Faculty of Nursing and Health Sciences, Nord University, Bodø, Norway; 2 Department of Physiotherapy, Nordland Hospital Trust, Bodø, Norway; 3 Department of Physiotherapy, Kongsgården Physiotherapy, Bodø, Norway

**Keywords:** multiple sclerosis, employment, work, work challenges, self, identity, vocational rehabilitaion

## Abstract

**Introduction:**

Despite mild disability, people with multiple sclerosis (PwMS) often face work-related challenges and reduced employment. Their experiences regarding work challenges are understudied.

**Purpose:**

To explore experienced work challenges and possibilities for sustained employment among employed PwMS with mild to moderate disability.

**Materials and methods:**

In-depth interviews with 26 employed PwMS analyzed using systematic text condensation guided by Goffman's theories on self and social interaction.

**Results:**

Three categories were developed: (1) unspoken work challenges, (2) the cumulative impact of mild MS symptoms on work challenges, and (3) disability and work challenges influence “who I am at work”. Communication about work challenges was often limited even when MS was disclosed. Most participants experienced difficulties related to cumulation and interplay of symptoms and work challenges, resulting in reduced work capacity, hours and negative thoughts about themselves, confidence and perceived prospects for job retention. Feeling capable in handling work tasks were believed to facilitate sustained employment.

**Conclusion:**

Mildly disabled PwMS often trivialized and avoided addressing work challenges to maintain self-concept, social order and role at work, even after MS was disclosed. Recognizing and addressing mild symptoms and work challenges while maintaining self-concept, sense of capability and acceptance at work, may facilitate sustained employment.

## 1 Introduction

Work challenges, reduced employment and early withdrawal from the workforce are common among people with multiple sclerosis (PwMS), even when disability is mild (1–3). These challenges are associated with various neurological symptoms, such as fatigue, reduced physical- and cognitive function, and psychological symptoms (2, 4, 5), as well as workplace-related factors (6). Unemployment, reduced positions, and work difficulties have substantial societal consequences (1, 3) and personal burdens, affecting such as finances (7), self-image (8), and health-related quality of life (9). Most employed PwMS have low levels of disability and workforce participation declines rapidly as disability progresses (1). Returning to the workforce can be challenging after experiencing skill depreciation, detachment from the labor market, and a shift in mindset toward inactivity can be challenging (10). A recent study indicated that early support may reduce work barriers for PwMS (11). This highlights the importance of understanding, managing and preventing impairments and work challenges while they are still minor and while individuals are still employed. Thus, it is important to understand work challenges and possibilities perceived by employed and mildly disabled PwMS to potentially facilitate sustained employment.

Reported work challenges include tasks that require motor and postural skills, and cognitive demands such as memory and stress tolerance, varying according to individuals' symptoms and occupations (6). Barriers related to the workplace include environmental factors such as noise, temperature, or stairs, as well as factors related to the social environment, such as interactional aspects and lack of understanding (6). Perceptions of stigma are reported in PwMS and mild disability, even though being more prevalent among people with more severe disability (12, 13). Stigma is for example reported as colleges' and employers' doubts about the PwMS' competence, which impact their confidence at work and, ultimately, their employment (14). MS-symptoms and changes in capabilities due to MS impact both thoughts about oneself and relationships with others (15). While these studies have provided valuable insights, much of the existing research on work challenges faced by PwMS is dated (6), and recent studies remain limited. In particular, few studies explore how these challenges are experienced when MS symptoms are mild or work-related difficulties are subtle, as most research includes PwMS across all levels of disability.

Studies on PwMS regardless of disability level have found that supportive and understanding coworkers, and effective communication with employers is important for job maintenance (6). Disclosure may be important for reducing stigma and discrimination (16, 17), reducing work barriers (6), and thus maintaining employment (16). However, PwMS report reluctance to disclose their diagnosis at work (16, 18, 19) because of concerns about the reactions of others (16, 19, 20) and the desire to maintain a public image (18). Nondisclosure prevents the identification of work barriers and adequate adjustments (6). It is therefore interesting to identify the experiences of PwMS with mild disability who are employed, have disclosed their diagnosis at work, and are participating in a work and health intervention that includes communication with employers. Such new knowledge can provide a better understanding of barriers related to work when disability is mild and can contribute to further develop support for PwMS aiming for sustained employment. Therefore, the aim of this study was to explore the experiences of employed PwMS with mild to moderate disability focusing on MS-related challenges and opportunities in the workplace.

## 2 Materials and methods

### 2.1 Design

A qualitative design using individual, in-depth, semistructured interviews was chosen. This design can provide deep insights into participants' first-hand experiences, perspectives and reflections (21, 22). This study was inspired by a phenomenological hermeneutic approach and anchored in the interpretative tradition (23), which acknowledges lived experiences and individuals' own descriptions and interpretations as sources of knowledge (22, 23).

### 2.2 Context of the study

To obtain empirical data on experiences of work challenges and possibilities from people who were still in the work force, this study was nested in a two-armed pilot feasibility randomized controlled trial (RCT) of employed PwMS with mild to moderate disability (EDSS: 0–3.5) (*N* = 29) (11). The trial explored a new intervention, CoreDISTparticipation, which aims to optimize sensorimotor function, physical activity and employment. The intervention was conducted in northern Norway and lasted for 10 weeks. It consisted of 1) a work-related consultation with an MS nurse and a consultation with a physiotherapist at the MS outpatient clinic that focused on balance, 2) one group-based indoor and one outdoor physiotherapy and high-intensity interval training in the municipality each week, and 3) a digital meeting on work adaptations with the MS nurse, the physiotherapist and the participant's line manager. The intervention aimed to facilitate and encourage communication between each participant and his or her line manager regarding aspects of employment such as potential needs. The control group received a physiotherapy consultation followed by usual care. Further details are provided in Table 1.

**Table 1:** Descriptions of CoreDISTparticipation (11).

CoreDISTparticipation Consists of three interlinked phases (a-b-c):	
a) MS outpatient (MS-OP) clinic (in addition to regular consultations)	
Day 1	- *A digital consultation with the MS nurse* (20 min) addressing work-related issues to promote participation in employment. A structured guide was used with the following themes: knowledge of MS in the workplace, work-related challenges experienced due to MS, potential needs and facilitators. - *A physiotherapy session* (60 min) exploring the potential for changes in balance and walking with the aim of focusing on possibilities and motivating the participant.
b) Municipality	
Weeks 2–5	- *A physiotherapy physical assessment* (60–90 min) that continued the exploration of the patient's impairments and potential for improvement. - *A digital meeting* (20 min) with the patient, physiotherapist, MS nurse and employer to set goals and discuss potential needs for adaptations regarding work and physical activity. - *Physiotherapist-led indoor sessions for four weeks using GroupCoreDIST^a^ exercises* in physiotherapy-led groups of 3–5 participants, 60 min × 2/week, addressing prerequisites for balance and walking. - The participants were encouraged to be physically active and to perform CoreDIST exercises at home. They had access to training videos on an open website (https://www.nord/CoreDIST).
c) Municipality	
Weeks 7–10	- *Physiotherapist-led outdoor sessions* in groups of 3–10, 60 min × 2/per week for five weeks to address prerequisites for balance through GroupCoreDIST exercises and high-intensity walking or running. - The participants were encouraged to continue communication with their employer. - The participants were encouraged to be physically active and to perform CoreDIST exercises at home. Reminders and encouragement for performing CoreDIST exercises based on training videos were sent to the participants' smartphones after each outdoor session.
In addition, the intervention included physical measurements, activity measurements with activity monitors and a questionnaire.	
**The control group** received a physiotherapy consultation at the MS-OP clinic followed by usual care in the municipality. Both groups continued regular consultations and medications.	

a GroupCoreDIST: Group-based training focusing on core (trunk) activation combined with DISTal functional movement; D, dual task, dose (high); I, individualized, insights; S, stability, somatosensory stimulation, selective movements; T, teaching, training.

### 2.3 Sample

All 29 PwMS who participated in the pilot RCT (intervention *n* = 15, control *n* = 14) were invited for the interview study to ensure rich material and to obtain a variety of perceptions. Recruitment for the RCT was conducted from the MS outpatient clinic at the Nordland Hospital Trust (NHT) with the following inclusion criteria: (1) diagnosed with MS in accordance with the McDonald criteria (24); (2) Expanded Disability Status Scale (EDSS) score (25) ≤3.5 (obtained from the participants' medical journals at the NHT); (3) ≥18 years of age; (4) employed (10%–100% full-time equivalent); and (5) registered at the MS outpatient clinic at the NHT. The exclusion criteria were as follows: (1) pregnant or retired at enrollment; (2) exacerbation within two weeks prior to enrollment; or (3) other serious conditions that compromise balance, walking or work capacity. Twenty-six people gave their informed consent to participate in the interview study, while three people declined due to a busy schedule (intervention *n* = 15, control *n* = 11). By inviting this sample, we aimed to capture the current employment experiences of individuals considered to have high potential for sustained work due to minor disability (1) and ongoing workforce participation (10), including some who had experience with supported communication with their employer.

### 2.4 Data collection

All the interviews were conducted by the first author via theme-based interview guides (Supplementary Material). The guides are the same as those used in a previous study (26). They were developed based on relevant literature on MS, work, methodology, and the researchers' experiences in neurological physiotherapy and occupational health. The researchers' experience helped shape the inclusion of topics and questions, focusing on perceived barriers and facilitators for employment, work situation, health and functioning, and work-related follow-up. A pilot interview with an individual with MS who met the inclusion criteria for the study without participating led to minor adjustments in the structure and length of the interviews. For example, some questions were moved from the main questions to follow-up prompts, the order of some questions were adjusted to improve the narrative flow, and general health questions were replaced with more targeted items related to work. related The questions were open-ended and asked about positive, negative and neutral experiences. Follow-up questions were asked, and the participants' responses were occasionally rephrased or summarized by the interviewer to confirm meaning and ensure communicative validation (21).This process involved reflecting participants' statements back to them in the interviewer's own words, allowing participants to affirm, clarify, or correct the interpretation, thereby strengthening the credibility of the data.

The interviews were conducted between November 2021 and June 2022. Participants from the intervention group were interviewed within two months after the pilot RCT to capture recent experiences and avoid recall bias. Participants from the control group were interviewed within seven months after the pilot RCT. All but one interview was conducted in person in an undisturbed office at NHT, Nord University, or the participants' workplaces. At the request of one participant, the interview was held digitally through a secure platform. All interviews were audio-recorded and ranged from 51 to 97 min each. Additionally, field notes were recorded after each interview to collect immediate or summarized impressions, such as “did not want to be treated as a sick person or talk about disease with leader” and “strongly emotionally affected”. These notes contributed to validating the recontextualized data following decontextualization, ensuring that it remained grounded in the original context (27).

### 2.5 Analysis

The interviews were transcribed verbatim by the first author and an assistant who was not involved in the study and were analyzed using systematic text condensation (STC), which was developed by Malterud (27). This pragmatic cross-case analysis is suitable for studies that aim to explore experiences, perceptions, and meanings from the perspective of the participants. The analysis was systematic, iterative and dynamic and involved decontextualizing and recontextualizing, searching for the essence of the phenomenon (27). This is in line with the phenomenological hermeneutic perspective (22) and the strategy described by Malterud (27). Given the aim of the study, no distinction was made between the intervention group and the control group in the analysis.

In line with the analytical strategy of systematic text condensation (27), the analyses comprised four steps: **1) Total impression:** A general, overall impression was obtained by reading all the interviews and fieldnotes to create a wider analytic space (27). MGH and ECA read all 26 interview transcripts, while BN read a considerable portion. After rereading and noting preliminary themes from the first 16 interviews, no new themes were identified, which led us to conclude that the sample and data were adequate (27). The last 10 interviews contributed meaning units that were included in the existing themes. The preliminary themes were discussed, and six preliminary themes related to the research question were agreed upon as starting points for organizing the data. **2) Identifying and sorting meaning units:** Text fragments containing information about the research question (i.e., meaning units) were identified by MGH and EA. Meaning units related to the preliminary themes were categorized and organized into code groups by MGH using NVivo software by QSR International, version 14. The interpretation, organization and classification of the meaning units and code groups were flexible and were continually discussed and adjusted to improve accuracy as the analysis and text evolved. **3) Condensation**: Subgroups within the code groups were discussed and agreed upon, and a condensate, i.e., a short first-person summary (27), was written for each subgroup by MGH and discussed with ECA. The names and borderlines of the code groups were flexible and were continually discussed and adjusted according to the evolving understanding (27). **4) Synthesizing:** The decontextualized summaries were synthesized and recontextualized into analytic texts in third person, presenting the most salient content and meaning about the phenomenon in the material of all participants, as described by Malterud (27). Some of the quotes that illustrated the findings were included. All the authors discussed and critically reviewed the analytic texts and interpretations. To validate the analysis and ensure that the analytic texts reflected the original context (27), they were compared to the full transcripts. On the basis of the analytic texts, three categories were identified that served as subheadings for the Results section. Finally, we returned to the full transcripts and searched for data that might challenge our conclusions. The themes, code groups, subgroups, categories and examples of meaning units are presented in Table 2.

**Table 2:** Overview of the steps in the analytic process, including preliminary themes, examples of meaning units, final code-groups and subgroups, and final categories.

Systematic, iterative and dynamic process of decontextualizing and recontextualizing 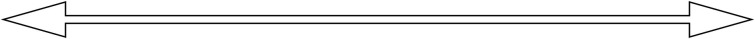				
Step 1: Total impression	Step 2: Identifying and sorting meaning units		Step 3: Condensation	Step 4: Synthesizing
Preliminary themes	Meaning unit (examples)	Code groups	Subgroup	Final category
Dialog and relations at work Denial Understanding	I think it might be a bit personal to go into that (…) Because, really, the employer has nothing to do with the diagnosis at all	Communication about work challenges at work	Dialog and openness	Unspoken work challenges
	You feel like a fool, plain and simple. But I think I've done the right thing by being open about it, the MS. Telling them exactly how it is. I've spoken to my bosses and said, unfortunately, I'm not a fool, that's just the way it is.		Understanding and misunderstanding	
	No, we talk very little about it. I don't want to focus so much on my health, because I'm quite capable.		Focus and denial	
Fatigue and tiredness	I have a bit of trouble with my hand, so I have a bit of a problem controlling the mouse. It can take on a life of its own, and I don't have the right feeling. So sometimes I have a bit of trouble getting the arrow to be exactly where I want it.	Work functioning	Physical function and capacity	The cumulative impact of mild MS symptoms on work challenges
Function and affection at work	I had to manage external meetings, right, and suddenly I couldn't get the words out.		Cognitive and psychological function and -capacity	
	I just can't keep going all the time. I can't keep going all day thinking and working and talking and doing things.		Fatigue and total capacity	
	In a way, it's who you are. So the job defines you in a way.	Meaning of work, work challenges and possibilities	Identity and value	Disability and work challenges influence “who I am at work”
Significance of work (Function and affection on work)	I'm not sure if I trust my left hand, because I have to use both hands there. I haven't tried and I've decided that I shouldn't try until I know that I have full control (…) then someone else will just have to do it.		Work ability and confidence	
	Sometimes I can be a bit like you: “Ugh, what now, what do I do now?” and I feel that this is going to be a crisis and that I'm going to lose my job.		Doubts and worries	

#### 2.5.1 Theoretical framework for interpretations of findings

On the basis of the empirical material, Goffman's theories on social interaction (28–30) were chosen to interpret and obtain a deeper understanding of the participants' experiences of work challenges and possibilities in relation to social interactions at work and meaning for the individual. Goffman's theories were considered relevant because they could contribute to understanding aspects of the participants' experiences, such as social interactions, stigma, and self-concept in relation to disability and work challenges.

Goffman's perspectives draw from the social constructionist and interactionist tradition (31) and emphasize that interactions and the individual's performances validated by others constitute an individual's self (30). Goffman claims that individuals construct an image of themselves on the basis of how others perceive and interact with them (28). The self and performance are constrained to be congruent with the mutually accepted “line”, “face”, and role to affirm the moral and ritual order of social life (29). The “*line*” is the expression of the evaluation of self and others in a social encounter and is shaped by the norms, roles, and hierarchies present in the interaction (29). The term “*face*” refers to the presented positive image of self that others may share, the way we want to be perceived and recognized by others, and the image we seek to maintain in interactions (29). Goffman argues that face is *maintained* when the line projects an image of self that is internally consistent and supported by others' judgments and is not challenged by actions or events that threaten this image (29). However, when the performance deviates from the line or role, such as a person with MS who suddenly cannot remember the topic of the conversation, the person is considered to be in “*wrong face*”. When maintenance of the positive publicly accepted image of self is not possible, the social face will be withdrawn leading to “*lost face*” (29), for instance due to impairments and work challenges. Motivated to maintain the line and the ritual order, individuals conduct *face-work* to maintain face and promote a particular conception of self and social processes (29, 30). This involves designating actions that are consistent with face and counteracting events that threaten face (29), which can influence the way challenges are expressed and manifested at work. Resources and the possession of traits and attributes that are considered desirable by others lead to a respectable self-image (32), whereas attributes that deviate negatively from expectations lead to stigma because these individuals are not considered “normal” (28). Goffman describes different types of stigma related to both body and character, inferred from such as disorders or unemployment (28). He claims that individuals with stigmatized attributes are expected to accept the standards of “normals”, underplay the significance of their difference, and voluntarily avoid situations in which “normals” find it difficult to show acceptance (28). Goffman emphasizes that the social meaning of impairment does not come from the impairment itself but from society and interactions (28). This theoretical perspective is relevant for understanding the experiences of employed PwMS regarding work challenges because their impairments may interfere with their social interactions and roles at work, even when their disability is mild.

### 2.6 Research team and reflexivity

The authors of this study are female physiotherapists with various backgrounds and experiences. MGH has experience in municipal health care and occupational health. ECA, BN and MS are clinical specialists in neurological physiotherapy with experience in specialist and municipal health care and research. Together they developed CoreDISTparticipation. In the RCT project BN served as the project leader and ECA contributed as a researcher.

Authors' backgrounds, experiences, knowledge and interests influence all phases of a research process. Therefore, we continuously attempted to be aware of our preconceptions and discussed our assumptions to ensure quality and trustworthiness (24). For example, we actively expanded our focus from emphasizing physical aspects to including psychosocial dimensions, ensuring that our interpretations were grounded in the full scope of participants' experiences. Moreover, the findings were discussed with researchers who were not involved in the study. These researchers had backgrounds in fields such as physiotherapy, nursing, and medicine, with expertise in research on MS, stroke, rehabilitation, and public health. These diverse and specific insights allowed for deep, relevant, and multiple discussions of interpretations on the basis of both proximity and distance (33).

### 2.7 Ethical considerations

This study was approved by the Regional Committees for Medical and Health Research Ethics in North Norway (REK North: 174837) and ethical authorities (data protection officer) at Nordland Hospital Trust. It was conducted in line with the Declaration of Helsinki and the Vancouver recommendations. All the participants signed confirmed written consent to participate. The interviewer (MGH) had no prior relationship with the participants, reducing potential bias and ensuring participant comfort. Participants were informed that MGH was a PhD candidate researching experiences with work-related barriers and facilitators, the intervention, and regular follow-up. The protection of privacy and personal data was ensured in compliance with all relevant rules and regulations and treated with care and respect. The Consolidated Criteria for Reporting Qualitative Research (COREQ) checklist (34) was used to ensure the quality of reporting for the study.

## 3 Results

The study included 26 participants: 20 females and six males, with a mean age of 48.5 years (range 31–66) and a mean EDSS score of 1.73 (range 0–3.5). Thirteen participants held full-time positions, while the remaining thirteen worked part-time. Most part-time workers held positions ranging from 50% to 69%, with disability benefits compensating for the remaining income, typically amounting to approximately 66% of previous earnings (35). The majority of participants had limited work participation at the time of the intervention and received some degree of financial support, such as sick pay, work assessment allowance, or disability pension. Occupations represented in the sample spanned a range of sectors, including healthcare, social services and education (e.g., teachers, healthcare workers, auxiliary nurses), administrative and office work (e.g., consultants, secretaries, managers), and manual work and service (e.g., cleaners, shop assistants, hairdressers, machine operators). All participants had disclosed their MS diagnosis to their employer, line manager, and most of their colleagues. Detailed participant characteristics are presented in Table 3.

**Table 3:** Participant characteristics based on data collected at the time of the intervention in the pilot-RCT (11).

Participant characteristics (*n* = 26)	
Variable	Value
Intervention-group, *n*	15
Control-group, *n*	11
Sex (male/female), *n* (%)	6 male/20 female (23%/77%)
Age at intervention, mean (SD) range	48,5 (8,36) 31–66
Age 30–39	3
Age 40–49	12
Age 50–59	7
Age 60–69	4
**Type of MS**	
RRMS, *n*	25
PPMS, *n*	1
EDSS, mean (SD) range	1.73 (0.99) 0–3.5
Social status	
Married, *n*	11
Cohabitant, *n*	6
Living alone, *n*	9
Education	
Longest completed education:	
High school, *n*	16
Bachelor or equivalent, *n*	9
Master or equivalent, *n*	1
Employment	
Full-time position, *n*	13
Part-time position, *n*	13
10%–49% position, *n*	2
50%–69% position, *n*	9
70%–90% position, *n*	2
On sick-leave/work assessment allowance, *n*	7
Degree of sick-leave: 50%–69%, *n*	3
Degree of sick-leave: 70 –89%, *n*	0
Degree of sick-leave: 90%–100%, *n*	4
Disability benefit, *n*	11
Degree of disability benefit: 30%–49%, *n*	2
Degree of disability benefit: 50%–69%, *n*	7
Degree of disability benefit: 70%–89%, *n*	1
Degree of disability benefit: 90%–100%, *n*	1
Occupations in healthcare/social services and education, *n*	9
Occupations in administrative/office work, *n*	11
Occupations in manual work and service, *n*	7

The analysis generated three categories: “unspoken work challenges”, “the cumulative impact of mild MS symptoms on work challenges”, and “disability and work challenges influence ‘who I am at work’”. These are described below and supported by some examples of relevant quotes from the participants.

### 3.1 Unspoken work challenges

All the participants had disclosed their diagnosis at work, with most of them emphasizing openness about the disorder. Many described a good relationship with their line manager, who had invited them to reach out if needed. However, several expressed no need or desire to discuss their MS-related challenges and “private matters” at work. Many described themselves as “quite healthy”, “lucky”, and “not very affected compared to others”, and several characterized their challenges at work as minor. Nevertheless, the majority described several MS-related symptoms that led to a variety of work-related challenges and reduced working hours.

Several individuals described ignoring symptoms and denying their disease, noting that they kept it at a distance and refused to let it define or limit them. Others described a process of realizing and accepting their disease and limitations. This process involved an internal struggle between what they wanted to do and what they could do, including who they wanted to be and who they were. Many described the desire to appear strong, normal, and able and stated that they would rather focus on their strengths and abilities at work than their MS-related challenges. One participant stated,

“There's something about when you're at work you want to be in that role, right. You want to function, you want to be part of something, and not something weak.” Rachel. EDSS: 2.0.

The importance and challenges of being understood were common themes in the material. Several participants described a fear of being misunderstood by others who might think they were lazy when they were tired, stupid when they forgot words, drunk when they were unsteady, or uninterested in social contact when they had difficulty dealing with noise. Some said that openness and explanations of their behavior prevented misunderstanding. They said openness felt better than pretending everything was fine and that being themselves at work facilitated their job retention. However, some individuals described difficulties in addressing work challenges due to the risk of appearing weak or pitiful and being misunderstood or overemphasized. Invisible symptoms such as pain, cognitive impairments, mental challenges, and bladder or bowel problems were said to be particularly uncomfortable to discuss and generated feelings of vulnerability. A few participants stated that they pretended to be healthy at work to avoid appearing ill despite knowing that it would make it more difficult for others to understand their actual problems. A participant remarked,

“I think openness is generally important, but at the same time, you kind of get those looks, like, ‘Oh my God, poor you.’ And you don't want that (…). We all want to appear as our best selves, in a way, when we're around others”. (Michell) EDSS: 1.0.

A few participants highlighted improved communication with their line manager due to participation in the CoreDIST intervention. This was particularly directed at the open discussions regarding work challenges that facilitated understanding, managers' knowledge about MS and support. Some of these participants said that the presence of health professionals in the meetings helped facilitate these elements. They emphasized that the improved understanding, knowledge and support led to better utilization of their resources and perceptions of improved possibilities for sustained employment. In contrast, other participants had found meetings with their manager regarding work challenges both during and prior to the intervention to be a waste of time. Some said their managers already understood their challenges. Others felt that the meetings were unhelpful and produced no concrete outcomes or even perceived the discussions as negative, uncomfortable and embarrassing and noted that they generated feelings of weakness and vulnerability. Some of the participants in the intervention said that the involvement of health professionals in the dialogs with the managers was experienced as uncomfortable because others intervened in work-related relationships or made the challenges appear more severe.

### 3.2 The cumulative impact of mild MS symptoms on work challenges

Although most participants characterized themselves as mildly affected by MS, they described a variety of interwoven symptoms. These included symptoms such as fatigue, altered sensibility, pain, depression, and difficulties with balance, vision, and memory. These symptoms were perceived as interacting with and reinforcing each other. They were also reinforced by workplace factors such as demanding tasks, workload, stress, noise, unpredictability, and social interactions. Most participants described that these symptoms and workplace factors individually or collectively affected their work capacity and ability and had led to changes in job roles, tasks, sick leave and/or reduced work hours. One participant stated,

“It's not that I want to stop working by any means, but I'm so tired, and I'm bothered so much in everyday life with, well, the cognitive and physical challenges. MS causes a lot of pain, nerve pain around the body”. (Roger) EDSS: 2.5.

The majority of the participants highlighted fatigue as their main problem, which led to reduced working hours and difficulty performing tasks. Fatigue was experienced as physical, cognitive, or total exhaustion, with many participants facing a combination of these. It was commonly attributed to MS symptoms (e.g., pain, numbness or vision impairments) compounded by factors in the work environment or job demands. These included bright lights, noise, responsibilities, appointments, stress, changes, and tasks that were cognitively or physically demanding. Several participants noted that fatigue reinforced other symptoms and work challenges, such as reduced concentration, and created a cycle of increased fatigue and difficulty performing work tasks.

Most participants experienced fatigue, reduced capacity and difficulties when performing physically demanding tasks such as lifting, carrying, walking, or prolonged standing or sitting. They also faced challenges with tasks that required fine motor skills such as computer work and laboratory or precision work with instruments. Consequently, they described inability or lower quality in the performance, or the need for modifications, assistance, or breaks. A participant who worked with her hands described frustrations and a sense of abnormality regarding somatosensory disturbances:

“I'm aware of things that you're not really aware of. Some other people just do those things with their hands, and it's completely normal. But I feel it suddenly and become aware of it, and that in itself feels a bit, well, like crap”. (Lisa) EDSS: 2,0.

Workplace factors were not only experienced to reinforce MS symptoms and work challenges, but they could also contribute to reduced symptoms, utilization of resources, and favorable conditions for job retention. Some participants experienced resources, capability, and reduced symptoms when performing sedentary work tasks, while others experienced this in physically active and varied tasks. Experiences of capability were described as being able to manage work demands or tasks, or to perform them with a level of quality or speed considered sufficient by the individuals themselves, their managers, or their colleagues. Some participants described capability and good work capacity when they completed one task at a time and focused on well-known and concrete tasks. These experiences were described as important for sustaining their employment. The majority described challenges related to cognitive difficulties, including issues with memory, concentration, attention, and problem solving. Many participants described problems with following procedures, writing documents, presentations or interacting with others. Additionally, many struggled with depression or anxiety. Cognitive and mental difficulties were described as increasing fatigue and reducing quality and efficiency at work, which made them feel uncomfortable, stressed and frustrated. Nearly half of the participants described difficulty performing new, fast, or multiple tasks, especially in the presence of noise and interruptions.

### 3.3 Disability and work challenges influence “who I am at work”

Several of the participants described their profession, work, or abilities at work as part of their identity, saying, “The job defines you” and “It's who you are”. Job retention was often emphasized as important, and work challenges and potential job loss raised concerns that their exacerbation could jeopardize the participants' future employment, identity, and sense of worth. A participant questioned,

“Who am I without my job? What am I worth then? (…) Then I kind of lose a lot of my identity”. (Nina) EDSS 1,5.

Some described work as an arena where they previously had been competent, coping, high-positioned and up-and-coming. However, due to MS-related challenges, this had changed. They expressed that struggles and lost capabilities at work felt like a loss or defeat of their self-expectations, and their image of themselves at work had changed. This involved negative reflections about themselves and feelings of being weak, ill, incompetent, unpredictable, and stupid. These reflections and feelings resulted in grief, discomfort and doubts about themselves and their work abilities. Several described concerns that their challenges affected the quality of their work. Some described a fear of making mistakes in critical situations involving safety, health, or finances, which undermined their confidence. One participant stated,

“I used to be a good (profession), but I'm not today. (….) I don't jump in anymore. I don't take it on straight away. I'm reluctant. Because of the challenges I have, the whole situation slows down. I'm a bit afraid of taking responsibility and jumping in. Before, that wasn't a problem”. (Frank) EDSS: 0.

Other participants described concerns regarding whether their work challenges affected their colleagues and managers. One participant shared strong negative feelings and a sense of letting colleagues and managers down when he was unable to perform well, particularly compared to younger, more skilled colleagues who adapted quickly to new digital tools. These perceptions made his MS stand out more clearly for him and contributed to his decision to leave the workforce.

Unpredictability was reported to be challenging for both employers and employees and often led to frustration and tiredness as well as anxiety about when work difficulties might suddenly arise. Several participants found it difficult to plan their work, both daily and over longer periods, owing to fluctuations in energy and functionality. However, during good days and periods, several participants reported being able to perform well at work. Despite facing work challenges, several participants felt that they possessed valuable competence in their field, which they believed was beneficial and contributory. Some participants mentioned that utilizing their resources at work fostered feelings of being capable in handling work demands, feeling normal, and being useful, which helped them stay motivated and employed.

## 4 Discussion

Three main findings were prominent. (1) Participants often trivialized or did not express MS-related work challenges to maintain their roles and desired appearance at work. (2) The accumulation and interaction of mild MS symptoms, combined with work characteristics, environmental and social elements, were perceived as essential work barriers. (3) The job was emphasized as defining who the participants were, and work challenges put their self-image, identity, and job retention at stake. Using their resources at work stimulated feelings of being capable in their roles and was perceived as important for sustained employment. These findings call for further interpretations within a framework that allows for a deeper understanding of the interrelated physical, psychosocial and contextual aspects. This will be discussed in the following section.

### 4.1 Maintaining self-concept and a desired appearance

The participants' trivialization and avoidance of expressing MS-related symptoms and work challenges seemed related to a desire to meet preferences and expectations regarding their abilities and roles at work. It is well documented that PwMS often conceal their disease (16, 18, 19). This concealment is often driven by fears of being seen as disabled or fraudulent, or concerns about being treated differently or pitied (18). Concealing disability and invisible symptoms may provide an opportunity to hide a disabled identity and maintain a perceived or projected image of self (18). However, the current study reveals that the participants avoided expressing MS-related symptoms and work challenges, even when their identity as disabled was known. This indicates that the desire to maintain a professional image and role may lead to hidden or trivialized work challenges even after disclosure. Additionally, participants' descriptions point to a connection between this avoidance and experiences or fear of stigma, which is supported by other studies (18, 19). However, previous studies have also found that low severity of disability (13) is related to lower perceptions of stigma, and that disclosure could lead to reduced stigma at work (17). This contrasts with our participants, who had a disclosed diagnosis and low EDSS scores. These findings indicate that fear and perceived stigma may lead PwMS to withhold information about work challenges, even at low levels of severity. Furthermore, the findings suggest that work challenges are not necessarily handled only by disclosing the diagnosis or by simply encouraging open communication.

In light of Goffman's theories about presentations of self, interactional order and stigma (28–30), the findings regarding avoidance and trivialization can reflect an effort to maintain a desired perception of self and social presentation. This effort aims to uphold social order and the impression of being part of the “normals”. The PwMS in our study who faced work challenges can be understood as being in conflict with what Goffman described as the mutually accepted “line”, “face” and “role” (29) because they could not present their projected or desired image of self. Thus, the participants' avoidance of expressing work difficulties can be seen as a form of what Goffman refers to as face-work (29). This face-work allows a person to maintain face and social order, to appear “normal” or as expected in work roles. It also avoids the risk of stigma resulting from their altered capabilities, as it may not be validated or desirable to others and deviate from social expectations and norms (28). Additionally, individuals who are stigmatized are expected to underplay the significance of their differences (28), and thus may explain the trivialization found in this study. While Goffman's theories offer valuable insights into social interaction and stigma, they may be concidered to not fully capture the evolving norms related to disability and inclusion, given their mid-20th-century origins. Nevertheless, recent research indicates that stigma and exclusion related to disability remain relevant (13, 19). Goffman's distinction between the “normals” and the “stigmatized” may, however, oversimplify the nuanced and dynamic aspects. When challenges are minor or fluctuate, as in people with MS and low disability, individuals may shift between visibility and invisibility, or between perceived “normalcy” and “difference”. Even so, his concepts resonate with the participants' descriptions of their experiences and perceptions in this study.

For some, trivializing challenges, avoiding discussions about work difficulties, and appearing “normal” may be beneficial strategies that prevent that information inconsistent with the line comes to light. Some participants described feeling uncomfortable, vulnerable, or weak when they discussed work difficulties with their managers, may indicate a sense of lost face. However, for most of the participants in the current study, avoidance *inhibited* understanding and *increased* the risk of losing face. It also contributed to stigmatization by employers and colleagues, as the lack of dialog often led to misunderstandings. In contrast, open dialog was emphasized as a positive strategy, leading to understanding, reduced need to pretend, and perceptions of being capable at work. These factors were said to facilitate job retention and satisfaction. In addition, face-work strategies such as avoidance or pretending, likely require effort and may contribute to increase fatigue, which most participants identified as their main issue. On this basis, the current study expands the knowledge base by emphasizing the importance of communication regarding not only the MS diagnosis, as previously reported (6, 16), but also work challenges. Such communication can increase understanding of the experienced work challenges and possibilities, leading to reduced stigma, better support, and an increased ability to manage one's needs at work. It can also enable individuals to utilize their capabilities, resulting in increased opportunities for sustained employment. While openness has advantages, it is important to consider risks and individual preferences in the specific context. Furthermore, most of the participants in the current study avoided expressing or trivialized their challenges even when they were encouraged and supported to address them. Thus, this study suggests that it is crucial for managers and health professionals to not only emphasize the importance of communicating about work challenges but also to ensure that this communication and the management of work challenges are conducted in a manner that maintains face. This approach requires managers and health professionals to possess knowledge about MS and demonstrate sensitivity to the experiences, needs, reflections, and desires of PwMS regarding work and self-presentation.

Most participants had partly withdrawn from the workforce or from previous positions at work. This aligns with previous studies showing that reduced employment is common among PwMS with low levels of disability (1, 2). The participants in the current study described that leaving their job or role at work negatively affected their identity and sense of value. In light of Goffman's theories, withdrawal may be another way to maintain perceptions and presentations of self as a type of face-work to prevent an anticipated threat to face or as a result of lost face or stigma (28, 29). Stigmatized individuals are expected to avoid situations where “normals” find it difficult to show acceptance (28). However, unemployment may not be validated or desirable in the eyes of others (28). Thus, both engagement and withdrawal may threaten self-concept and the presentation of self, resulting in considerations regarding employment or unemployment even at low levels of disability. According to Goffman, face-saving maneuvers are shaped by game-like calculations, in which individuals determine whether there is more to gain than to lose by these maneuvers (29). Thus, an employee's decision to hide, minimize, expose, or withdraw is likely to depend on individual calculations about maintaining face, social order, and acceptance. These preferences and decisions are influenced by contextual and interactional aspects, such as social norms and the validation of others (29, 30). This is further supported by more recent research, which highlights that communication is directly related to the psychosocial work environment (16). However, Goffman's theories tend to underemphasize structural factors such as organizational frameworks and policy environments, which also shape individuals' preferences and decisions regarding face-saving maneuvers and work participation. For example, comprehensive and generous welfare systems may facilitate decisions to withdraw from the workforce, since the economic disparity between working and receiving welfare benefits is minimal. Additionally, strong employment protection legislation may influence individuals' willingness to be open about work challenges, either by encouraging disclosure or, conversely, by enabling them to avoid openness and continue working despite reduced productivity. Thus, the current study highlights the importance of addressing these considerations and calculations prior to withdrawal from work, and especially addressing the psychosocial work environment in the management of work challenges among PwMS. This study suggests the need to emphasize an inclusive and adaptive work environment where employees feel valued and accepted regardless of their disability.

### 4.2 Little streams make big rivers

The findings indicate that work challenges appear to be influenced by the accumulation and interplay of workplace-related barriers and symptoms, even when these symptoms are characterized as mild and disability levels are low. Previous studies have identified a range of individual and environmental work barriers regardless of disability level (4–6), and that PwMS often describe one particular problem impacting job retention (36). In contrast, the current study suggests that barriers and symptoms are not experienced as discrete or isolated but as dynamically interconnected and mutually reinforcing, even when disability is mild. These findings raise questions about the characterization of the severity of disability and the impacts of this characterization. PwMS with mild disability may feel expected to be content with their relatively low level of disability, knowing they face fewer challenges than other PwMS with more severe disability. At the same time, they may fear being seen as complainers. Moreover, previous studies have noted that PwMS often fear and face disbelief from others, such as health professionals and people at work, about invisible or vague symptoms because they appear healthy (18, 20). This disbelief leads to PwMS doubting their own legitimacy (18), and their seemingly good functioning leads to their needs not being explored or met (18). In light of the findings and interpretations of this study, we suggest that it is crucial for employers, health professionals and PwMS to recognize the severity of interacting and accumulating symptoms and work challenges, as these can significantly affect the ability to maintain employment. The findings highlight the importance of considering the combination of both physical, cognitive, and environmental aspects in the management of work challenges. Fatigue is especially relevant because it has been emphasized as the primary reason for reduced employment and a significant factor in work challenges in both the current and previous studies (1, 8). In the current study, fatigue appeared related to the accumulation of symptoms and challenges, as the participants often mentioned several difficulties that individually were mild or vague but that each one contributed to fatigue. Moreover, fatigue appeared to interact with other symptoms and work challenges in a reinforcing cycle. For instance, it was described as worsening cognitive difficulties, which in turn further intensified the experience of fatigue. These findings indicate that the experiences of fatigue are complex and multifaceted. This underscores the importance of understanding and addressing the aspects contributing to fatigue in each individual with MS, particularly in relation to their specific work challenges.

Findings in the current study indicate that MS-related challenges at work put job retention, self-perception, self-image and self-confidence at stake, which aligns with previous studies on PwMS (8, 14). However, the current study suggests that these aspects are relevant even for PwMS with low EDSS scores and only minor work challenges, and influence their considerations of their work and employment. This relevance applies not only to current experiences but also to feared future impacts. The participants' descriptions indicate that work challenges negatively influenced their capable and confident selves and impacted their employment. Conversely, feeling capable in work tasks was perceived as important for sustained employment. Drawing on Goffman's theories, these findings suggest that work capabilities and challenges are related to self-concept and identity. Moreover, Goffman adds the interactional perspectives in this context, proposing that self is constituted by performances that are validated by others and is based on how others perceive and interact with the individual (28, 30). The relevance of these perspectives for employment was highlighted in a study by Kirk-Brown and Van Dijk (37) who found that employer's recognition of PwMS's capability may promote the employees' long-term plans for maintenance of employment. Thus, the current study suggests that work challenges that are characterized as minor have a substantial impact on essential concepts of self, identity and employment for PwMS. Moreover, it highlights the importance of facilitating experiences of capability at work that are perceived and recognized by both the individual and by others at work. Such recognition, as an individual with resources and capabilities, may be facilitated by reasonable adjustments to work requirements and the work environment. This appears relevant based on several participants' experiences, which highlighted the influence of workplace factors on symptoms, utilization of resources, and conditions for job-retention. The adjustments may include changes to elements such as work tasks, workload, timeframes, disturbances, and aspects of the social environment. However, it is important to consider the employee's perspective and ensure their sense of maintained face when addressing and introducing work adjustments.

### 4.3 Strengths and limitations

A strength of the current study is the use of in-depth interviews with PwMS, which led to a deep and comprehensive understanding of the relationships among the various aspects related to the experience of work challenges. By including 26 employed PwMS, this study obtained a rich sample of experiences from different contexts and insights into work challenges prior to unemployment. However, the inclusion of only employed PwMS who participated in a pilot RCT can be seen as a limitation because the experiences of unemployed and nonparticipating PwMS may also have provided valuable insights. Participation in the intervention and interviews may have increased awareness of work challenges as well as opportunities and resources because both perspectives were emphasized. Another limitation is the gender imbalance in the sample, with few male participants. Gender norms and expectations may influence how work challenges are experienced, expressed, and managed. For instance, emotional and relational reflections may be more commonly or differently articulated by women. As a result, the findings may reflect gendered patterns, and future research should aim for more balanced gender representation to explore potential differences. Nevertheless, the gender difference reflects the composition of the MS population.

Despite these limitations, the application of theories from the social sciences strengthened the study by enriching the interpretations with perspectives that extend beyond individual symptoms to include social meanings, norms, and interactions that influence work participation. Although dated and somewhat limited in nuance, these theories still illuminate contemporary and relevant issues. The use of these theories also provides theoretical generalizability and transferability of the findings and allows us to move beyond individual findings to wider significance and applicability (38). The findings may have transferability to other conditions with low disability and risk of unemployment, because the influence and importance of perceptions and presentation of self in work may be relevant regardless of diagnosis. However, some aspects of this study may be more relevant to countries with comprehensive welfare systems that offer generous benefits and social support, such as those in Scandinavia. These systems can influence individuals' considerations regarding openness about work challenges and decisions related to work participation.

## 5 Conclusion

This study revealed that employed PwMS often trivialize or avoid expressing symptoms and work challenges, and struggle to maintain their roles at work, even when MS is disclosed, disability is mild, and dialog is offered. This appearance may be related to efforts to avoid stigma by maintaining face and social order. However, such efforts may instead lead to adverse impacts on stigma, face, and role at work due to reduced understanding and support from colleagues and managers. PwMS particularly experience the accumulation and combination of symptoms and workplace-related aspects to have substantial impacts on possibilities for productive and satisfactory workdays and sustained employment, putting identity at stake. Feeling understood, accepted, and experiencing a sense of capability at work appeared beneficial for maintaining face, job satisfaction, and perceived possibilities for sustained employment. Such feelings may be facilitated by open communication about mild MS symptoms, work challenges, and resources, while maintaining the individual's self-concept. Therefore, addressing both individual and social aspects, as well as other workplace-related aspects of work challenges and possibilities, is necessary to facilitate sustained employment for PwMS with mild to moderate disability.

## 6 Implications

The knowledge from this study may encourage health professionals, employers, and PwMS to recognize and address work challenges, capabilities and opportunities even when disability is low. Health professionals and employers should be aware of the vulnerable situation that PwMS may face at work and therefore discuss and address work challenges in a manner that PwMS maintain their desired self-perception and presentation. One approach can be to offer a proactive and understanding communication, offering work situations individualized to promote experiences of capability, as well as a supportive and accepting psychosocial work environment. Thus, the study calls for an integrated and multifaceted approach to facilitate sustained employment for PwMS with low levels of disability.

This knowledge can potentially contribute to reducing work challenges, increasing positive work experiences, and ultimately improving job retention for PwMS when the disability level is still low. Further research is needed to determine how to approach, communicate about, and reasonably accommodate work challenges while maintaining employees' desired self-perception and presentation at work.

## Data Availability

The datasets presented in this article are not readily available because the ethics committee has not provided permission to share the data. Requests to access the datasets should be directed to maria.g.hartvedt@nord.no.
