# Supplementary material for: Mild multiple sclerosis challenges shape work experiences, affect self-concept, and are often trivialized despite disclosure
Source: Front Rehabil Sci. 2025 Nov 12;6:1677114. doi: 10.3389/fresc.2025.1677114 (PMC12646985; doi:10.3389/fresc.2025.1677114)
Supplement: Supplementary file 1 [file Table1.docx]

Supplementary Material

**Interview guide for people with MS (intervention group)*** Questions that were less central to the present study.

| Introduction | Thank you for your willingness to participate in this project and be interviewed here! You have now been through 10 weeks of training in the CoreDISTparticipation project and attended meetings with the MS nurse, physiotherapists, and a meeting with the MS nurse, physiotherapist and employer all together where the topic was work. This interview will be about how you have experienced being at the meetings, your work situation and how the project has affected it.  Do you have any questions before we start? | | |
| --- | --- | --- | --- |
| Topic | Thematic/research questions | Possible interview questions | Follow-up questions |
| Opening question | Open question after participating in CoreDISTparticipation. | What has it been like for you to be involved in this project? |  |
| Work situation and health | How does the participant experience his/her own health and work situation? What does the participant think affects the job situation? What does the participant think about barriers and facilitators to his/her own participation in work now and in the future? What does the participant think about possible connections between his/her perception of the job situation and health status? | Can you describe what you do in your job? What is a normal working day like?  What does the job mean to you?  How does MS affect your working day and your participation in work?  Does it lead to any challenges/barriers?    What adaptations do you need or have been made in your work/workplace?  What works well in your work?  How is the relationship with your line manager?  How does being/not being physically active affect your working day and your participation in work?* | % employed/sick leave, etc. When and why did you reduce? Are there any consequences of working reduced hours? Any benefits? How would it be for you to work more?  What are the benefits of working? What are the disadvantages?  What do you think about that? Is there anything psychological/cognitive/emotional that affects your working day? Is it different now versus before you got MS? How do you experience it?  In what way? How do you feel about that? What could have been done differently to reduce these challenges? What else would make it easier for you to be at work? Have these challenges been less at any point? If so, why? What do you think about these challenges in the future? What will be important then?  How does it work? How do you feel about that? Do you make any adjustments yourself? What could be done to improve or facilitate the workday?  What conditions do you think are the basis for it working so well? What resources do you have that you would use more of in your work?  How does it affect opportunities and challenges in your everyday work? How are your relationships with colleagues? Who knows that you have MS? What does it mean that others know/don't know?  How is it addressed/discussed at work? What are the opportunities for PA during work hours?* |
| Experiences from the training in the intervention* | How did the participant experience the training in the intervention, and the relevance for work?* | How has the training in the intervention affected you in your job?*  Was there anything you hoped would be affected that wasn't? Expectations?* | Is there anything different now after the training? How does it override to working life? Positive/negative? How does it feel? Is this something you want to continue with? Why/why not? Has it affected other factors?*  What do you think is needed to achieve that?* |
| Experiences from the meeting with the MS nurse related to the work situation and the disease | What kind of reflections did the participant make after the meeting with MS nurse at the beginning of the period? How did the participant experience the digital meeting? | Can you describe the work-related meeting with the MS nurse?  What was it like for you to be part of this meeting?    What did it mean for the meeting to be digital?  Were there any particular elements you thought were particularly good? Any elements that should have been changed?  What do you think about the usefulness of the meeting?  What were you left with after the meeting? What was the result? | What did you talk about? What did you think about that?  What was positive, what was challenging or negative? How were you met by the MS nurse? How did you perceive the adjustment to you and your situation was ensured? How were your perspectives considered? What role did you have? The others?  Why do you think this was good/not good?  Why/why not?  Past, present, and future. |
| Experiences of the meeting with the MS nurse, physiotherapist and line manager | How did the participant experience the digital meeting with MS nurse, physiotherapist and line manager at work, and what kind of reflections has the participant made? | Can you describe the work-related meeting?  What was it like for you to participate in this meeting?  What did it mean for the meeting to be digital?  How did it feel to talk about this in the presence of your line manager?  Were there any particular points you thought were particularly good? Any points that should have been changed?  What thoughts were you left with after the meeting?  What was the meaning and usefulness of the meeting? | What did you talk about? What did you think about that?  What was positive, what was challenging or negative? How did you perceive the adjustment to you and your situation was ensured? How were your perspectives considered? What role did you have? The others?  What did it mean for you and for your everyday work life?  Why do you think this was good/not good?  Past, present, and future.  Why was it useful or meaningful/ why not? What relevance will this have for the future? |
| Experiences of completing a form to evaluate the goals that were set | What was the participant’s experiences with the evaluation form? | What is your experience of the evaluation form at the end of the period?  How did you complete the form (alone, together with your manager)?  What was it like for you to complete the form?  What thoughts did you have about the goals?  What dialog did you and your line manager have about this form?  What did you get out of the evaluation?  How do you think this will be followed up in the future? | What thoughts did you have about it?  What impact have these goals had? How have they been followed up? Were new goals set?  What have you talked about? What was it like for you to talk about it?  What do you think is needed to benefit from it?  What will it mean to you? |
| The road ahead | What reflections does the participant have on his/her own situation in the future and the development of services for people with MS | What do you think about your job situation in the future?  What will it take for you to continue working?  Have you considered other types of jobs?  Have you made any reflections about what participation in CoreDISTparticipation may have done in terms of thoughts about work and participation in work?  Are there other professionals who should have been involved?  Suggestions for improvements? | Quantity? Type? Accommodations? Support? Own prerequisites? |
| Closure |  | Is there anything you would like to share about your participation in the 10 weeks of CoreDISTparticipation that we haven't talked about?  How did you experience being interviewed? |  |

**Interview guide for people with MS (control group)*** Questions that were less central to the present study.

| Introduction | Thank you for your willingness to participate in this project and be interviewed here! You have been a participant in the control group of this research project that is looking at a new follow-up for people with MS, where working life has been the topic. This interview will focus on your experiences, perceptions and thoughts about your working life and your everyday working life.  Do you have any questions before we start? | | |
| --- | --- | --- | --- |
| Topic | Thematic/research questions | Possible interview questions | Follow-up questions |
| Work situation and health | How does the participant experience his/her own health and work situation? What does the participant think affects the job situation? What does the participant think about barriers and facilitators to his/her own participation in work now and in the future? What does the participant think about possible connections between his/her perception of the job situation and health status? | Can you describe what you do in your job? What is a normal working day like?  What does the job mean to you?  How does MS affect your working day and your participation in work?    Does it lead to any challenges/barriers?  Who do you talk to about work-related challenges?  What adaptations do you need or have been made in your work/workplace?  What works well in your work?    How is the relationship with your line manager?  What would it be like if your employer knew more about MS and common challenges with MS?  What do you think should be the basis for you to have the best possible working day and be able to participate in work as much and as long as possible? | % employed/sick leave, etc. When and why did you reduce? Are there any consequences of working reduced hours? Any benefits? How would it be for you to work more?  What are the benefits of working? What are the disadvantages?  What do you think/feel about that? Is there anything psychological/cognitive/emotional that affects your working day? Is it different now versus before you got MS? How do you experience it?  In what way? How do you feel about that? What could have been done differently to reduce these challenges? What else would make it easier for you to be at work? Have these challenges been less at any point? If so, why? What do you think about these challenges in the future? What will be important then?  In what contexts is it being addressed? What has been said? How do you feel you are met and understood?  How does it work? How do you feel about that? Do you make any adjustments yourself? What could be done to improve or facilitate the workday?  What conditions do you think are the basis for it working so well? What resources do you have that you would use more of in your work?  How does it affect opportunities and challenges in your everyday work? Relationships with colleagues? Who knows that you have MS? What does it mean that others know/don't know?  What benefit could it have? What disadvantages could it have? |
| Experiences of physical activity and the impact of function on everyday work and work participation.* | What experiences does the participant have with physical activity and its impact on work?* | Can you describe your physical activity in daily life?*  How does being/not being physically active affect your working day and your participation in work?*  What are you doing/are you doing anything else to maintain or improve your resources to be able to function as well as possible in working life?* | Type, frequency, duration, intensity?*  Positive/negative/none? How is PA addressed/discussed at work? What are the opportunities for PA during working hours?* |
| Experiences from meetings with MS nurse related to the work situation and the disease | What kind of experiences and reflections does the participant have from the regular follow-up meetings with the MS nurse? | Do you usually have meetings with the MS nurse at the hospital?  Can you tell me about any instances where work has been a topic in your conversations with the MS nurse?  Can you tell me about any instances where physical activity has been a topic in your conversations with the MS nurse?  Who would you like to talk about this with? | How often? When? What do you talk about? How do you experience it? What does it mean to you?  How did you experience it? What has it led to? What has it meant?    Why? Others? |
| Experiences from follow-up by healthcare professionals or others. | What follow-up does the participant have? What benefit does it have for working life? Reflections on what could be useful in terms of developing the services. | Do you have or have you had follow-up in the last year by healthcare professionals or others?  Is there anyone you would like more follow-up from? | Which one? Frequency? Content? Why/why not? What does it mean to you and for your everyday work life?  Why? If so, when do you think it might be relevant? |
| The road ahead | What reflections does the participant have on his/her own situation in the future? | What do you think about your job situation in the future?  What will it take for you to continue working?  Have you considered other types of jobs? | Quantity? Type? Accommodation? Support? Own prerequisites? |
| Closure |  | Is there anything you'd like to share about working life that we haven't talked about?  How have you experienced being interviewed? |  |
